# Preoperative patellofemoral anatomy affects failure rate after isolated patellofemoral inlay arthroplasty

**DOI:** 10.1007/s00402-020-03651-9

**Published:** 2020-10-30

**Authors:** Matthias J. Feucht, Patricia M. Lutz, Conrad Ketzer, Marco C. Rupp, Matthias Cotic, Andreas B. Imhoff, Jonas Pogorzelski

**Affiliations:** 1 Department for Orthopedic Sports Medicine, Technical University Munich, Ismaninger Str. 22, 81675 Munich, Germany; 2 Department of Orthopaedics and Trauma Surgery, Medical Center, Faculty of Medicine, Albert-Ludwigs-University of Freiburg, Freiburg, Germany

**Keywords:** Patellofemoral arthroplasty, Malalignment, Patellar maltracking, Patella alta, TT–TG, TT–PCL, Trochlear dysplasia

## Abstract

**Purpose:**

To analyze whether preoperative patellofemoral anatomy is associated with clinical improvement and failure rate after isolated patellofemoral arthroplasty (PFA) using a modern inlay-type trochlear implant.

**Methods:**

Prospectively collected 24 months data of patients treated with isolated inlay PFA (HemiCAP^®^ Wave, Arthrosurface, Franklin, MA, USA) between 2009 and 2016, and available digitalized preoperative imaging (plain radiographs in three planes and MRI) were retrospectively analyzed. All patients were evaluated using the WOMAC score, Lysholm score, and VAS pain. Patients revised to TKA or not achieving the minimal clinically important difference (MCID) for the total WOMAC score or VAS pain were considered failures. Preoperative imaging was analyzed regarding the following aspects: Tibiofemoral OA, patellofemoral OA, trochlear dysplasia (Dejour classification), patellar height (Insall–Salvati index [ISI]; Patellotrochlear index [PTI]), and position of the tibial tuberosity (TT–TG and TT–PCL distance).

**Results:**

A total of 41 patients (61% female) with a mean age of 48 ± 13 years could be included. Fifteen patients (37%) were considered failures, with 5 patients (12%) revised to TKA and 10 patients (24%) not achieving MCID for WOMAC total or VAS pain. Failures had a significantly higher ISI, and a significantly lower PTI. Furthermore, the proportion of patients with a pathologic ISI (> 1.2), a pathologic PTI (< 0.28), and without trochlear dysplasia were significantly higher in failures. Significantly greater improvements in clinical outcome scores were observed in patients with a higher preoperative grade of patellofemoral OA, ISI ≤ 1.2, PTI ≥ 0.28, TT–PCL distance ≤ 21 mm, and a dysplastic trochlea.

**Conclusion:**

Preoperative patellofemoral anatomy is significantly associated with clinical improvement and failure rate after isolated inlay PFA. Less improvement and a higher failure rate must be expected in patients with patella alta (ISI > 1.2 and PTI < 0.28), absence of trochlear dysplasia, and a lateralized position of the tibial tuberosity (TT–PCL distance > 21 mm). Concomitant procedures such as tibial tuberosity transfer may, therefore, be considered in such patients.

**Level of evidence:**

Level III, retrospective analysis of prospectively collected data.

## Introduction

Patellofemoral arthroplasty (PFA) has become a valid treatment option for relatively young and active patients with isolated patellofemoral osteoarthritis (OA) [26, 42, 52]. Compared to total knee arthroplasty (TKA), PFA offers the advantage of sparing healthy bone, cartilage, and ligaments, thereby preserving native knee kinematics [10, 14, 44]. In a recent randomized controlled trial comparing PFA and TKA for isolated patellofemoral OA, PFA resulted in better range of motion and better patient-reported outcome [44]. However, relatively high reoperation and revision rates remain an issue of debate [5, 34, 51, 58]. Whereas implant design-specific complications were the main reasons for failure with early PFA designs, progression of tibiofemoral OA is considered the main failure mode of contemporary used implants [5, 51].

Another important reason leading to failure especially in the early postoperative course is unaddressed patellar maltracking [2, 17, 43, 51]. Given the complex interaction between dynamic muscle action, passive soft-tissue restrains, surface geometry of the patellofemoral joint, and limb alignment, patellofemoral maltracking is commonly seen as a multifactorial problem [20, 22, 28]. In the native knee, patella alta and a lateralized tibial tuberosity are well-accepted risk factors for painful patellofemoral maltracking and instability [19, 22, 46, 47]. Although a treatment algorithm to address tibiofemoral and/or patellofemoral malalignment in combination with PFA has been proposed [27], the relevance of these parameters in patients undergoing PFA remains largely unknown and warrants further investigation [2].

It is generally accepted that patient selection is the key for successful PFA. Better knowledge of preoperative risk factors for unsatisfactory outcomes may improve survival rates after PFA. The purpose of this study was to analyze whether preoperative patellofemoral anatomy is associated with clinical improvement and failure rate after isolated PFA using a modern inlay-type trochlear implant. The hypothesis was that patella alta and a lateralized tibial tuberosity are associated with less clinical improvement and higher failure rates after inlay PFA.

## Materials and methods

Prospectively collected clinical outcome data were retrospectively analyzed to study the association between preoperative anatomy of the patellofemoral joint and clinical improvement as well as failures after isolated inlay PFA.

Between 2009 and 2016, a consecutive series of 109 patients were treated with inlay PFA at the authors’ institution. Surgery was indicated in patients with disabling patellofemoral OA or chondrosis refractory to conservative treatment [23, 26, 27]. Contraindications were symptomatic tibiofemoral OA with pain during activities of daily living, systematic inflammatory arthropathy, chondrocalcinosis, chronic regional pain syndrome, active infection, and fixed loss of knee range of motion [23, 26, 27].

Preoperative evaluation consisted of a thorough patient history, clinical evaluation of the affected knee, plain radiographs in three planes, and magnetic resonance imaging (MRI) in all patients. Additional weight-bearing full-leg radiographs and computer tomography scans were obtained in patients with suspected abnormal limb alignment. Based on the findings of the preoperative evaluation, patients were treated with either isolated inlay PFA or combined inlay PFA, as described in a previously published algorithm [27]. In patients undergoing combined PFA, concomitant procedures such as MPFL reconstruction, distal femoral osteotomy, or high tibial osteotomy were performed to address patellofemoral instability or limb malalignment.

**Fig. 1:**
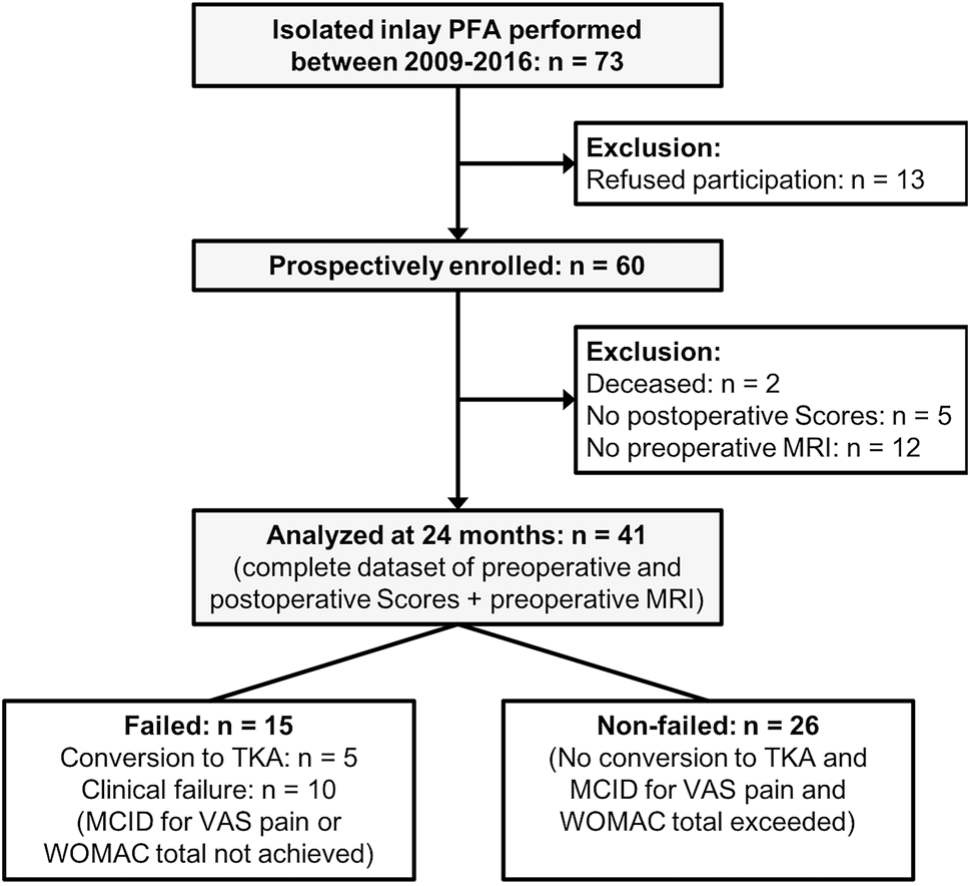
Flowchart of the patient selection and evaluation process. Abbreviations: PFA, patellofemoral arthroplasty; *n* = number of patients; MRI, magnetic resonance imaging; TKA, total knee arthroplasty; MCID, minimal clinically important difference; VAS, Visual analog scale; WOMAC, Western Ontario McMaster Universities Osteoarthritis Index)

For the purpose of this study, only patients undergoing isolated inlay PFA were considered for inclusion. Additional inclusion criteria were: Availability of digitalized preoperative plain radiographs in three planes (antero-posterior, lateral, and skyline views at 45° knee flexion) and MRI, and the availability of complete preoperative and follow-up scores, as described below. Exclusion criteria were previous soft-tissue or bony procedures at the ipsilateral knee affecting patellofemoral anatomy, absence of preoperative imaging, and metal implants in the knee area with artifacts on MR images or motion artifacts. A flowchart of the patient selection and evaluation process is shown in Fig. 1.

### Surgical technique and postoperative rehabilitation.

All patients were treated with the HemiCAP^®^ Wave Patellofemoral Resurfacing System (Arthrosurface, Franklin, MA, USA) according to the recommendations of the manufacturer. The resurfacing system incorporates a cobalt chrome trochlear component that is connected to a titanium bone anchoring fixation stud via a taper interlock, and an all-polyethylene patellar component. Eight different implants with varying offsets and radii of curvature allow for a patient-specific geometry match. A lateral parapatellar approach without eversion of the patella was used. Patelloplasty and circumpatellar denervation were performed in all patients; however, the patella was not routinely resurfaced. In our clinical practice, the patella is only resurfaced in patients with patellofemoral incongruence because of severe patellar dysplasia, focal osteonecrosis or osteolysis, and subchondral bone defects [27]. Patients performed partial weight-bearing with 20 kg for 2 weeks. Full range of motion was allowed immediately.

### Data collection

All patients were evaluated preoperatively and at 24 months postoperatively by a specially trained research assistant, who was not a participating surgeon (M.C.) during clinical follow-up visits. Clinical outcome was evaluated using the Western Ontario and McMaster Universities Osteoarthritis (WOMAC) score [4], Lysholm score [48], and visual analog scale for pain (VAS pain) [25]. The WOMAC score was assessed according to the KOOS User´s Guide (available at https://www.koos.nu/KOOSGuide2003.pdf). Five Standardized answer options were given as 5 Likert boxes and each question got a score from 0 to 4. A normalized percentage score (100 indicating no problems and 0 indicating extreme problems) was calculated for each subscale (pain, stiffness, function) and for the total score. Clinical improvement for all outcome measures was calculated as the difference between preoperative and follow-up scores at 24 months (delta, **∆**).

Preoperative imaging (plain radiographs and MRI) was analyzed regarding tibiofemoral/patellofemoral OA and anatomy of the patellofemoral joint. All radiographic measurements were performed independently by two orthopedic residents specifically trained in the measurements obtained in the present study. To determine the interobserver reproducibility, interclass correlation coefficients (ICCs) and Cohens Kappa were calculated.

The following measurements and classifications were performed:

*Tibiofemoral OA* Preoperative tibiofemoral OA was assessed on antero-posterior radiographs using the Kellgren–Lawrence grading scale [32]. Based on the severity of preoperative OA, patients were assorted to one of the two groups: Mild (grades 0–I) or moderate to severe (grades II–IV) [57].

*Patellofemoral OA* Preoperative patellofemoral OA was assessed on skyline view radiographs in 45° of flexion using the Iwano classification system [30]. Based on the severity of preoperative OA, patients were assorted to one of the two groups: Mild (grades 0–I) or moderate to severe (grades II–IV) [16].

*Trochlear dysplasia* The shape of the trochlea was categorized according to the Dejour classification on MRI using the 3 most proximal images demonstrating articular cartilage [18]. Patients were stratified based on the absence or presence of trochlear dysplasia (Types A–D) [16, 37].

*Insall–Salvati Index (ISI)* ISI was determined on lateral radiographs in 30° of flexion as the ratio between the patellar tendon length and the greatest pole-to-pole length of the patella [29]. Knees with a ratio > 1.2 were considered to have patella alta [7], and patients were assorted to one of the two groups: ISI ≤ 1.2 or > 1.2.

*Patellotrochlear index (PTI)* PTI was measured on sagittal MRI as described by Biedert and Albrecht [6]. The length ratio between the articular surface of the patella and the articulating trochlea was calculated. A ratio of < 0.28 was considered pathologic [7], and patients were assorted to one of the two groups: PTI ≥ 0.28 or < 0.28.

*Tibial tubercle–trochlear groove (TT–TG) distance* The TT–TG distance was measured on axial MRI images as the mediolateral distance between the midpoint of the insertion of the patellar tendon and the trochlear groove [19, 45]. A TT–TG distance of > 20 mm was considered pathologic. However, only one patient showed a pathologic TT–TG distance, wherefore no further grouping was performed.

*Tibial tubercle–posterior cruciate ligament (TT–PCL) distance* The TT–PCL distance was measured on axial MRI images as described by Seitlinger et al. [46] and defined as the mediolateral distance between the midpoint of the insertion of the patellar tendon and the medial border of the PCL tibial insertion. Based on a recent systematic review [9], a TT–PCL distance of > 21 mm was considered pathologic and patients were assorted to one of the two groups: TT–PCL ≤ 21 mm or > 21 mm.

For data analyzation, patients were classified as “failed” or “non-failed” PFA. The “failed” group consisted of patients undergoing conversion to TKA during the follow-up period and patients considered clinical failures, defined as not having achieved the minimal clinically important difference (MCID) for the total WOMAC score (10 points) or VAS pain (2 points), as reported for TKA in previous studies [13, 49]. “Failed” and “non-failed” patients were compared regarding the above-described preoperative measurements and classifications. Furthermore, improvements of clinical outcome scores (**∆** values) of all patients not undergoing TKA were analyzed regarding these parameters of interest.

### Statistical analysis

Statistical analysis was performed using SPSS software version 25.0 (IBM-SPSS, New York, USA). Continuous variables were calculated as mean ± standard deviation. Categorical variables were reported as count and percentages.

Normal distribution of all data was evaluated with the Kolmogorov–Smirnov test. Normally distributed continuous variables were compared using an unpaired two-sample *t* test. Non-normal distributed continuous variables and categorical variables were compared using the Mann–Whitney *U* test or Fisher’s exact test. The level of significance was set at *p* < 0.05.

To determine the interobserver reproducibility, intraclass correlation coefficients (ICC) were calculated for continuous variables. ICC values > 0.9 were considered excellent, values between 0.8 and 0.9 were considered good and values < 0.8 were considered poor. For categorial variables, Cohens Kappa was calculated and strength of agreement was rated as “almost perfect” (> 0.81), “substantial” (0.61–0.80), “moderate” (0.41–0.60), and “fair” (0.21–0.40) [33].

A post hoc power analysis was performed using G*Power 3.1 software (Franz Paul, Kiel, Germany). With an *α* of 0.05, a power of 0.85 to detect the MCID for the WOMAC score (10 ± 10 points) and VAS pain (2 ± 2 points) was calculated based on the included study population. The study was, therefore, sufficiently powered to test the hypothesis.

The study was approved by the Ethics Committee of the Technical University of Munich (Ethical approval no. 419/13).

## Results

A total of 41 patients (61% female) with a mean age of 48 ± 13 years and a mean BMI of 26 ± 3.5 kg/m^2^ could be included. Of those, 5 patients (12%) were converted to TKA during the study period, and 10 patients (24%) did not achieve MCID for WOMAC total or VAS pain at the 24-month follow-up (clinical failures). Therefore, 15 patients (37%) were classified as “failed”, and 26 patients (63%) were classified as “non-failed”.

ICC values for continuous variables were good or excellent for all measurements (ISI: 0.819, PTI: 0.955, TT–TG: 0.903, TT–PCL: 0.919). Kappa values for categorial variables were “almost perfect” for patellofemoral OA (0.941), and “substantial” for tibiofemoral OA (0.691) and trochlear dysplasia (0.749).

Patellar resurfacing was performed in 29%, whereas 71% did not undergo patellar resurfacing. No significant difference in failure rates (*p* = 0.003) or clinical outcome scores (*p* > 0.05) was observed between both groups (*p* = 0.003).

Comparison between preoperative and follow-up outcome scores of the total study group, the “non-failed” group, and clinical failures is summarized in Table 1. Significant improvements of all outcome scores were observed for the total study cohort and the “non-failed” group, whereas no significant improvement was observed in clinically failed patients. Furthermore, the “non-failed” group showed significantly higher absolute values at the 24-month follow-up and significantly higher **∆**-values for all outcome scores.

**Table 1:** Outcome scores of the total study group, the non-failed group, and clinical failures (patients not achieving MCID for WOMAC total or VAS pain at 24 months)

	Total study group	Non-failed	Clinical failures
VAS pain preoperative	5.6 ± 2.0	6.2 ± 1.8	4.0 ± 1.4^**b**^
VAS pain 24 months	2.9 ± 1.9^a^	2.6 ± 2.1^a^	3.8 ± 0.9^b^
VAS pain delta	2.6 ± 2.2	3.6 ± 1.9	0.2 ± 0.8^b^
WOMAC total preoperative	67.8 ± 13.6	65.7 ± 14.3	73.5 ± 10.2
WOMAC total 24 months	79.0 ± 15.3^a^	83.2 ± 13.4^a^	67.9 ± 14.9^b^
WOMAC total delta	11.2 ± 19.1	17.6 ± 17.7	−5.5 ± 11^b^
WOMAC pain preoperative	64.9 ± 18.6	60.8 ± 18.7	75.0 ± 14.5 ^b^
WOMAC pain 24 months	82.0 ± 15.4^a^	85.4 ± 15.5^a^	73.5 ± 12.0^b^
WOMAC pain delta	17.1 ± 22.2	24.6 ± 20.1	−1.5 ± 15.8^b^
WOMAC stiffness preoperative	59.3 ± 22.1	56.5 ± 23.1	66.3 ± 18.7
WOMAC stiffness 24 months	70.4 ± 22.1^a^	76.0 ± 18.7^a^	56.3 ± 24.5^b^
WOMAC stiffness delta	11.1 ± 31.1	19.6 ± 27.9	−10.1 ± 29.6^b^
WOMAC function preoperative	69.8 ± 14.1	68.2 ± 15.2	73.7 ± 10.4
WOMAC function 24 months	78.9 ± 15.8^a^	83.4 ± 13.9^a^	67.6 ± 15.1^b^
WOMAC function delta	8.4 ± 19.2	14.2 ± 19.1	−6.1 ± 9.2^b^
Lysholm preoperative	41.2 ± 18.2	40.2 ± 18.6	43.9 ± 17.8
Lysholm 24 months	65.3 ± 20.4^a^	69.1 ± 20.3^a^	55.3 ± 18.0^b^
Lysholm delta	24.0 ± 20.0	28.9 ± 21.2	11.4 ± 7.5^b^

Patients undergoing conversion to total knee arthroplasty were excluded from data analysis

Values are shown as mean ± standard deviation

*MCID* minimal clinically important difference, *VAS* Visual analog scale, *WOMAC* Western Ontario McMaster Universities Osteoarthritis Index

^a^Statistically significant improvement compared to preoperative (*p* < 0.05)

^b^Statistically significant difference compared to “non-failed” (*p* < 0.05)

**Fig. 2:**
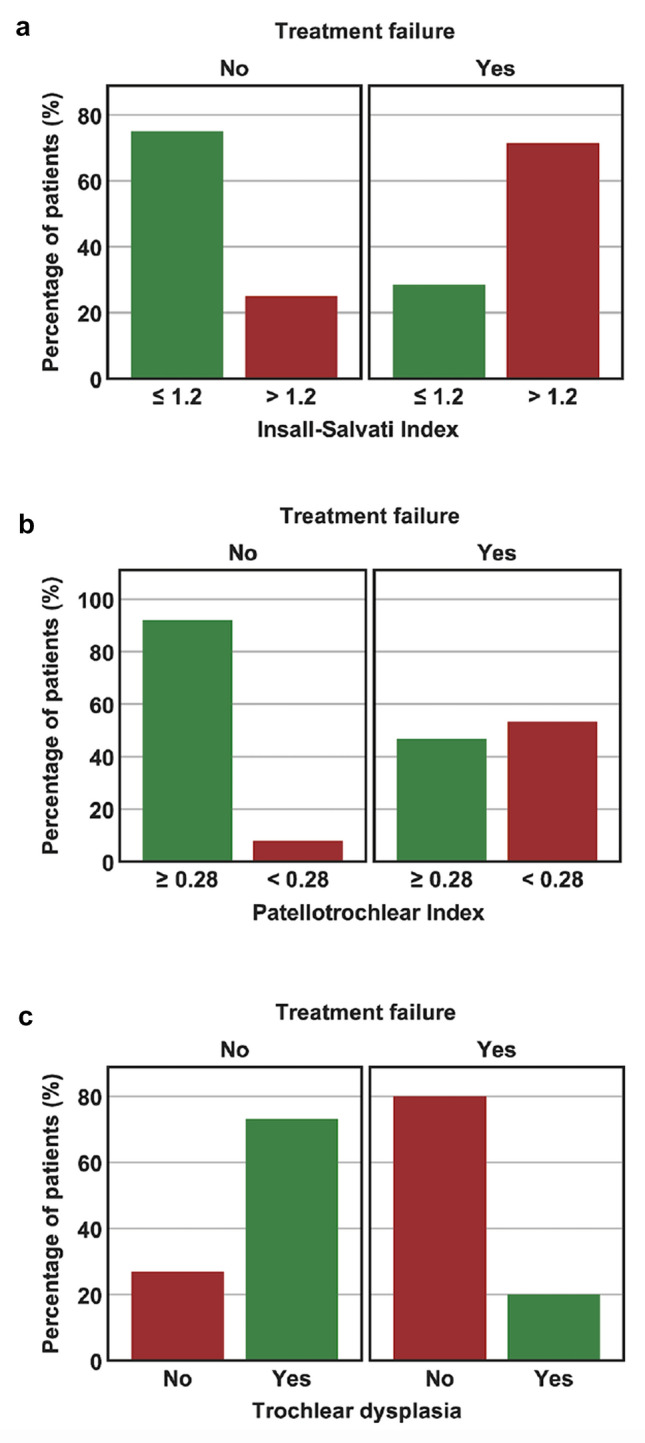
Comparison between “failed” and “non-failed” PFA. The group “failed” consisted of patients converted to TKA during the study period or not achieving minimal clinically important difference (MCID) for WOMAC total or VAS pain at 24 months. **a** The proportion of patients with a pathologic Insall–Salvati Index (> 1.2) was significantly higher in “failed” patients; **b** the proportion of patients with a pathologic Patellotrochlear Index (< 0.28) was significantly higher in “failed” patients; **c** the proportion of patients without trochlear dysplasia was significantly higher in “failed” patients

Comparison between “failed” and “non-failed” patients is summarized in Table 2. Statistically significant differences between both groups were found for age, ISI, PTI, and trochlear dysplasia. “Failed” patients were significantly older, had a significantly higher ISI, and a significantly lower PTI. Furthermore, the proportion of patients with a pathologic ISI (> 1.2), a pathologic PTI (< 0.28), and without trochlear dysplasia were significantly higher in “failed” patients (Fig. 2). With regard to trochlear dysplasia, 80% of “failed” patients had a normal trochlea, whereas 73% of “non-failed” patients showed a dysplastic trochlea (27% Type A, 27% Type B, 15% Type C, and 4% Type D).

**Table 2:** Comparison between “non-failed” and “failed PFA”

Variable	Group		*p* value
	Non-failed	Failed	
Gender			0.517
Female	17 (65%)	8 (53%)	
Male	9 (35%)	7 (47%)	
Age (years)	45.0 ± 13.1	53.5 ± 10.5	**0.038**^**a**^
BMI (kg/m^2^)	25.7 ± 3.6	26.8 ± 3.3	0.362
Tibiofemoral OA (Kellgren–Lawrence)			0.318
None or Grade I	15 (63%)	6 (43%)	
Grade II, III, or IV	9 (38%)	8 (57%)	
Patellofemoral OA (Iwano)			1.000
None or Grade I	8 (35%)	5 (39%)	
Grade II, III, or IV	15 (65%)	8 (62%)	
Insall–Salvati Index	1.1 ± 0.2	1.4 ± 0.2	**< 0.001**^**a**^
Insall–Salvati Index			**0.008**^**a**^
≤ 1.2	18 (75%)	4 (29%)	
> 1.2	6 (25%)	10 (71%)	
Patellotrochlear Index	0.5 ± 0.19	0.37 ± 0.18	**0.038**^**a**^
Patellotrochlear Index			**0.002**^**a**^
≥ 0.28	23 (92%)	7 (47%)	
< 0.28	2 (8%)	8 (53%)	
TT–TG distance (mm)	11.3 ± 3.9	13.6 ± 3.4	0.065
TT–PCL distance (mm)	19.9 ± 5.8	22.9 ± 4.0	0.092
TT–PCL distance			0.156
≤ 21 mm	13 (52%)	4 (29%)	
> 21 mm	12 (48%)	10 (71%)	
Trochlear dysplasia (Dejour)			**0.010**^**a**^
Normal	7 (27%)	12 (80%)	
Type A	7 (27%)	0 (0%)	
Type B	7 (27%)	1 (7%)	
Type C	4 (15%)	2 (13%)	
Type D	1 (4%)	0 (0%)	
Trochlear dysplasia (Dejour)			**0.001**^**a**^
Normal	7 (27%)	12 (80%)	
Types A–D	19 (73%)	3 (20%)	

The group “failed” consisted of patients converted to TKA during the study period or not achieving minimal clinically important difference (MCID) for WOMAC total or VAS pain at 24 months

Continuous variables are shown as mean ± standard deviation, categorical variables are shown as number of patients and percentages per group

*OA* osteoarthritis, *TT–TG* tibial tuberosity trochlear groove, *TT–PCL* tibial tuberosity posterior cruciate ligament, *mm* millimeters, *kg* kilograms, *kg/m*^2^ kilograms per square meter

^a^Statistically significant difference between both groups

Improvement of clinical outcome scores (**∆**-values) with regard to preoperative measurements is shown in Table 3. Statistically significant greater improvements were observed in patients with a higher preoperative grade of patellofemoral OA (Iwano grade ≥ II), ISI ≤ 1.2, PTI ≥ 0.28, TT–PCL distance ≤ 21 mm, and a dysplastic trochlea.

**Table 3:** Improvement of clinical outcome scores (delta **∆** between preoperative and postoperative values) with regard to parameters of interest

	∆ VAS pain	*p* value	∆ WOMAC total	*p* value	∆ Lysholm	*p* value
Tibiofemoral OA (Kellgren–Lawrence)		0.151		0.957		0.362
None or Grade I	3.0 ± 2.3		12.4 ± 17.5		22.4 ± 19.6	
Grade II, III, or IV	1.9 ± 1.9		12.8 ± 21.0		28.7 ± 19.4	
Patellofemoral OA (Iwano)		0.603		**0.046**^**a**^		0.146
None or Grade I	3.0 ± 2.2		1.1 ± 15.8		15.1 ± 22.0	
Grade II, III, or IV	2.5 ± 2.2		15.4 ± 18.8		26.8 ± 19.5	
Insall–Salvati Index		**0.017**^**a**^		**0.001**^**a**^		**0.010**^**a**^
≤ 1.2	3.4 ± 2.3		22.9 ± 14.9		33.8 ± 17.9	
> 1.2	1.6 ± 1.8		2.9 ± 17.0		16.9 ± 17.5	
Patellotrochlear Index		**0.014**^**a**^		0.200		**0.031**^**a**^
≥ 0.28	3.6 ± 2.2		15.6 ± 21.5		31.6 ± 22.2	
< 0.28	1.6 ± 1.7		7.1 ± 17.0		17.0 ± 15.8	
TT–PCL distance		**0.020**^**a**^		0.174		**0.014**^**a**^
≤ 21 mm	3.1 ± 2.1		14.0 ± 20.4		29.2 ± 21.1	
> 21 mm	1.3 ± 1.8		4.4 ± 15.7		11.5 ± 10.9	
Trochlear dysplasia (Dejour)		**0.026**^**a**^		0.060		0.058
Normal	1.9 ± 2.7		4.5 ± 20.5		17.0 ± 17.1	
Types A–D	3.2 ± 1.6		16.5 ± 16.6		29.6 ± 20.7	

*MCID* minimal clinically important difference, *VAS* Visual analog scale, *WOMAC* Western Ontario McMaster Universities Osteoarthritis Index, *OA* osteoarthritis, *TT–PCL* tibial tuberosity posterior cruciate ligament, *mm* millimeters, *kg* kilograms, *kg/m*^2^ kilograms per square meter

For data analyzation, non-failed and clinical failures (patients not achieving MCID for WOMAC total or VAS pain at 24 months) were included. Patients undergoing conversion to total knee arthroplasty were excluded

Values are shown as mean ± standard deviation

^a^Statistically significant difference

## Discussion

The most important finding of the present study was that preoperative patellofemoral anatomy is significantly associated with clinical improvement and failure after isolated inlay PFA. Less improvement and higher failure rates were observed in patients with patella alta (ISI > 1.2 and PTI < 0.28), absence of trochlear dysplasia, and a lateralized position of the tibial tuberosity (TT–PCL distance > 21 mm). Further important findings were that patients in the “failed” group were significantly older and that patients with a higher grade of preoperative patellofemoral OA (Iwano Grade ≥ II) demonstrated greater improvement of clinical outcome scores.

Treatment of isolated patellofemoral OA is still a matter of debate [24]. Although PFA has been used for more than 50 years [8, 41], it is still considered controversial [35, 40]. Inconsistent results and relatively high failure rates have led to a decline in popularity in the past [8, 11, 21]. Drawbacks of the implant design, especially of the trochlear component, are believed to be the major reason for failures with early implants [38, 39]. With the introduction of new implant designs, PFA has produced more consistent results and has regained importance in clinical practice [3, 26, 42, 52]. Currently available trochlear components can be divided into two groups: inlay and onlay designs. Inlay design trochlear components are implanted flush with the surrounding cartilage after creation of a bone bed within the native trochlea. Onlay design trochlear components completely replace the anterior compartment by using the same anterior cut as known from total knee arthroplasty. Clinical outcomes of both implant designs has been reported to be comparable. Nevertheless, a considerable number of patients fail and have to undergo revision to TKA [36]. The failure rate of 37% after a follow-up of 2 years observed in the present study is considerable higher compared to reported failure rates of modern-type PFA in other studies [36, 52]. However, most studies consider failure as revision to TKA only. The conversion rate to TKA of 12% observed in the present study is comparable to other studies [34, 36]. However, we also considered failure if the MCID for the functional outcome scores was not achieved at the 24-month follow-up. These more stringent criteria for the definition of failure may, therefore, explain the relatively high failure rate observed in the present study. This assumption is further strengthened by a study of Kazarian et al. [31]. In their series of 63 patients treated with primary isolated onlay PFA, less than 4% required revision surgery after a follow-up of approximately 5 years; however, fewer than two-thirds of patients were satisfied with the result [31]. Since 24% of patients in the present study were considered clinical failures but did not undergo revision to TKA, we strongly suggest to include unsatisfactory clinical results in future failure analysis.

A broad consensus exists that appropriate patient selection is the key to improve outcomes after PFA and there is growing interest in identifying preoperative predictors [15, 16, 26, 37, 57]. In the native patellofemoral joint, malalignment such as patella alta and a lateralized position of the tibial tuberosity can cause patellar maltracking with abnormal patellofemoral joint forces, leading to patellofemoral instability, anterior knee pain, and the development of cartilage degeneration [22, 28, 47]. Therefore, uncorrected patellofemoral malalignment may also adversely affect the outcome after PFA. In the present study, patella alta and a lateralized tibial tuberosity were associated with less clinical improvement and higher failure rates after isolated inlay PFA.

In the extended knee, the patella is located laterally but moves medially as it engages the trochlear groove [22]. Especially the lateral trochlear facet, which extends further proximal than the medial one, plays an important role in guiding patellar tracking during early flexion [22]. In knees with patella alta, this guiding mechanism is diminished, since the patella engages the trochlea not until higher flexion angles [1]. This has been shown to cause increased lateral patellar displacement and tilt, reduced contact area, and elevated joint stress [55, 56]. Several methods to measure patellar height on lateral radiographs have been described [7, 54]. The Insall–Salvati index was chosen for the present study since it has been shown to be the most reliable method [12, 53, 54]. However, a problem of patellar height measurement on lateral radiographs is that only bony landmarks are used and the true articular congruence between the patella and trochlea is not measured [6, 7]. For this reason, the Patellotrochlear index has been introduced, which measures overlap of the patellar and trochlear cartilage on sagittal MR images [6]. In the present study, both indices influenced clinical outcome. Patients in the “failed” group showed a significantly higher Insall–Salvati index and a significantly lower Patellotrochlear Index, indicating higher position of the patella in failed patients. Furthermore, patients with a pathologic Insall–Salvati index (> 1.2) or a pathologic Patellotrochlear index (< 0.28) was significantly overrepresented among failures. We, therefore, conclude that preoperative patella alta is a risk factor for failure after inlay PFA, and a concomitant distalization of the tibial tuberosity should be considered in patients with a pathologic Insall–Salvati index or Patellotrochlear index. Despite the known biomechanical alterations due to patella alta [55, 56], the observed association with worse results may also be related to the implant used in the present study. The femoral component of the HemiCAP® Wave is an inlay design which covers mainly the central part of the trochlea and does not have a superolateral extension to guide the patella in early flexion. Therefore, patella alta cannot be compensated by this specific implant. This problem has also been observed in another study by Beckmann et al. [2]. In their series of 20 patients treated with the HemiCAP® Wave prosthesis, 11 patients underwent revision surgery for recurrent pain and “clunk” phenomenon. The authors found a significantly increased modified Insall–Salvati index in the revised group as well as abraded areas craniolateral of the inlay implant. All patients were revised using an onlay-type prosthesis and pain as well as function improved postoperatively. The authors, therefore, concluded that inlay PFA should be considered contraindicated in patients with patella alta and that an onlay PFA system reaching further proximal should be considered [2]. It must be noted, however, that it remains unknown to what extend a larger implant can compensate for patella alta. In our opinion, distalization of the tibial tuberosity is a more anatomic approach. Further studies are needed to better define the appropriate management of patients with patella alta and symptomatic patellofemoral OA.

A lateralized position of the tibial tuberosity leads to lateral patellar tracking, elevated lateral patellofemoral joint contact pressure, and reduced patellar stability [47]. The position of the tibial tuberosity has usually been measured with the TT–TG distance [19, 45]. However, the TT–TG distance is confounded by several factors such as knee flexion, torsion of the femur, and trochlear dysplasia [9, 46]. Therefore, the TT–PCL distance has been introduced, which describes pure lateralization of the tibial tuberosity [9, 46]. Within this study, only one patient demonstrated a pathologic TT–TG distance of > 20 mm, whereas 22 patients (56%) had a pathologic TT–PCL distance of > 21 mm. Furthermore, patients with a pathologic TT–PCL distance experienced less clinical improvement in VAS pain and Lysholm scores compared to patients with a normal TT–PCL distance. In a previously published treatment algorithm to address tibiofemoral and/or patellofemoral malalignment in combination with PFA, the position of the tibial tuberosity was only assessed with the TT–TG distance, and medialization of the tibial tuberosity was recommended as a concomitant procedure in patients with a TT–TG distance > 20 mm [27]. However, the findings of the present study indicate that the TT–PCL distance should also be incorporated in the preoperative work-up. With regard to different implants, an onlay-type femoral component permits the surgeon to alter the position of the trochlear groove, allowing for small corrections of the TT–TG distance [50]. However, the TT–PCL distance can neither be corrected with an onlay design nor an inlay design. Based on the results of the present study, concomitant medialization of the tibial tuberosity should, therefore, be considered in patients with a TT–PCL distance > 21 mm, irrespective of the implant design.

Another important finding of the present study was that absence or presence of trochlear dysplasia on preoperative MRI scans predicted failure and clinical outcome. More specifically, 80% of patients in the “failed” group showed no evidence of dysplasia; whereas, only 27% in the “non-failed” group had a normal trochlea. Furthermore, patients without trochlear dysplasia demonstrated significantly less improvement in VAS pain scores. The association between the presence of trochlear dysplasia and better clinical outcomes has also been observed by other groups [15, 37], and it is believed that patients with patellofemoral OA secondary to trochlear dysplasia are less prone to develop degenerative changes of the tibiofemoral joint [15]. PFA should, therefore, be indicated with caution in patients without trochlear dysplasia. However, it must be noted that only few patients in the present study had a high-grade dysplasia, which may be difficult to treat with an inlay-type PFA.

Progression of tibiofemoral OA has been reported to be the most common failure mode after PFA, especially in studies with medium to long-term follow-up [5, 51]. In the present study, the grade of preoperative tibiofemoral OA was not associated with failure or less clinical improvement. However, the follow-up period of 24 months is most likely too short to address this issue, which was not the main intention of this study. In general, PFA is considered contraindicated in patients with preexisting tibiofemoral OA grade > II, especially if symptomatic [27, 57]. With regard to the severity of preoperative patellofemoral OA, the present study found that patients with only mild OA (Iwano grade ≤ I) experienced significantly less improvement of the WOMAC score compared to patients with moderate or severe OA (Iwano grade ≥ 2). This observation confirms the results of deDeugd et al. [16], who also found less improvement in pain and function after PFA in patients with only mild patellofemoral OA. Therefore, PFA should be indicated with caution in patients presenting with chondromalacia on MRI but only minimal evidence of patellofemoral OA on plain radiographs.

This study has several limitations. First, although clinical outcome data were collected prospectively, the study design was retrospective. Second, the number of patients analyzed was relatively small. However, isolated PFA is not very common and the number of patients is comparable to other studies. Furthermore, a post hoc power analysis demonstrated that the study was sufficiently powered. Third, of 60 enrolled patients, complete data (clinical scores, radiographs, and MRI) were only available for 41 patients. The follow-up rate is, therefore, only 70%. However, most patients were “lost” because of missing preoperative digitalized MRI (20%) and only 8% were lost because of incomplete clinical scoring. We believe that this circumstance lowers the risk for selection bias which is considerably higher if patients do not provide clinical outcome scores because of dissatisfaction. The clinical follow-up rate in the present study was 88%. Fourth, the follow-up period is probably too short to analyze the relevance of tibiofemoral OA progression. However, this was not the main intention of the present study. Fifth, this is a descriptive study and therefore, the causality of the observed anatomical differences cannot be proven.

## Conclusion

Preoperative patellofemoral anatomy is significantly associated with clinical improvement and failure after isolated inlay PFA. Less improvement and higher failure rates must be expected in patients with patella alta (ISI > 1.2 and PTI < 0.28), absence of trochlear dysplasia, and a lateralized position of the tibial tuberosity (TT–PCL distance > 21 mm). Patient selection is, therefore, critical to improve outcomes in the future and concomitant procedures such as tibial tuberosity transfer may be considered in patients with patella alta or a lateralized tibial tuberosity.
